# Altered Hypothalamic Functional Connectivity Following Total Sleep Deprivation in Young Adult Males

**DOI:** 10.3389/fnins.2021.688247

**Published:** 2021-10-01

**Authors:** Jing Qi, Bo-Zhi Li, Ying Zhang, Bei Pan, Yu-Hong Gao, Hao Zhan, Yong Liu, Yong-Cong Shao, Xi Zhang

**Affiliations:** ^1^School of Medicine, Nankai University, Tianjin, China; ^2^Department of Neurology, The Second Medical Center, Sleep Medicine Research Center, National Clinical Research Centre for Geriatric Diseases, Chinese PLA General Hospital, Beijing, China; ^3^The Eighth Medical Center of the General Hospital of People’s Liberation Army, Beijing, China; ^4^Air Force Medical Center, PLA, Beijing, China; ^5^National Clinical Research Centre for Geriatric Diseases, Second Medical Center, Chinese PLA General Hospital, Beijing, China; ^6^Brainnetome Center, Institute of Automation, Chinese Academy of Sciences, Beijing, China; ^7^National Laboratory of Pattern Recognition, Institute of Automation, Chinese Academy of Sciences, Beijing, China; ^8^CAS Center for Excellence in Brain Science and Intelligence Technology, Institute of Automation, Chinese Academy of Sciences, Beijing, China; ^9^School of Artificial Intelligence, University of Chinese Academy of Sciences, Beijing, China; ^10^Shool of Psychology, Beijing Sport University, Beijing, China; ^11^School of Biological Science and Medical Engineering, Beihang University, Beijing, China

**Keywords:** sleep deprivation, vigilant attention, hypothalamus, functional connectivity, fMRI

## Abstract

**Background:** Sleep deprivation can markedly influence vigilant attention that is essential to complex cognitive processes. The hypothalamus plays a critical role in arousal and attention regulation. However, the functional involvement of the hypothalamus in attentional impairments after total sleep deprivation (TSD) remains unclear. The purpose of this study is to investigate the alterations in hypothalamic functional connectivity and its association with the attentional performance following TSD.

**Methods:** Thirty healthy adult males were recruited in the study. Participants underwent two resting-state functional magnetic resonance imaging (rs-fMRI) scans, once in rested wakefulness (RW) and once after 36 h of TSD. Seed-based functional connectivity analysis was performed using rs-fMRI for the left and right hypothalamus. Vigilant attention was measured using a psychomotor vigilance test (PVT). Furthermore, Pearson correlation analysis was conducted to investigate the relationship between altered hypothalamic functional connectivity and PVT performance after TSD.

**Results:** After TSD, enhanced functional connectivity was observed between the left hypothalamus and bilateral thalamus, bilateral anterior cingulate cortex, right amygdala, and right insula, while reduced functional connectivity was observed between the left hypothalamus and bilateral middle frontal gyrus (AlphaSim corrected, *P* < 0.01). However, significant correlation between altered hypothalamic functional connectivity and PVT performance was not observed after Bonferroni correction (*P* > 0.05).

**Conclusion:** Our results suggest that TSD can lead to disrupted hypothalamic circuits, which may provide new insight into neural mechanisms of attention impairments following sleep deprivation.

## Introduction

Sleep deprivation is a common complaint in modern society. It seriously affects multiple cognitive processes, such as vigilant attention, work memory, and decision making (Krause et al., 2017). Vigilant attention is essential to complex cognitive processes and is especially susceptible to sleep deprivation (Collet et al., 2020). The attentional performance declines in a dose-response manner as sleep pressure increases depending on the non-linear interaction between circadian and sleep homeostasis processes. The longer the duration of sleep deprivation, the worse the accumulating attention impairments. Attention deficits could result in industrial disasters, medical errors, traffic accidents, and safety loopholes (Patanaik et al., 2015). Clarifying the neural mechanisms for attentional deficits would help us counter the negative effects elicited by sleep deprivation.

The human hypothalamus is a small subcortical region located below the thalamus. It comprises numerous nuclei and has extensive connections with the brainstem, basal nuclei, thalamus, limbic regions, and cerebral cortex, which plays a central role in sleep-wake regulation (Arrigoni et al., 2019). Distinct hypothalamic cell populations have been identified in the regulation of sleep-wake homeostasis. For instance, the suprachiasmatic nucleus (SCN) is the central circadian clock that maintains sleep-wake circadian rhythms on an approximately 24-h period (Venner et al., 2019). The activity of SCN changes in parallel with the sleep-wake rhythm and rapid eye movement (REM) sleep/non-REM (NREM) sleep cycle. The SCN neuronal activity exhibits a significant increase after NREM sleep deprivation (Deboer et al., 2003). Besides, orexin/hypocretin neurons in the lateral hypothalamus are wake-active while melanin-concentrating hormone neurons promote REM sleep (Yamashita and Yamanaka, 2017). An animal study revealed altered neuronal activity in rat hypothalamic regions after sleep deprivation, including the paraventricular nucleus, lateral hypothalamus, mammillary bodies, and arcuate nucleus (Fifel et al., 2018). Vargas and colleagues found that acute sleep deprivation influenced the human hypothalamic-pituitary-adrenal axis functioning, with elevated pre-stress cortisol levels but declined cortisol response to the stressors (Vargas and Lopez-Duran, 2017). Taken together, these findings suggest that the hypothalamic function is vulnerable to sleep restriction. However, few studies have focused on the alterations in hypothalamic functional coupling with other brain regions following sleep deprivation.

Beyond the sleep-wake regulation, the hypothalamus has also been implicated in attentional modulation since it is a major part of the arousal system (Jones, 2003). The lateral hypothalamus plays a substantial role in the attentional processing in associative learning by regulating the information processing in the amygdala central nucleus (Wheeler et al., 2014). Moreover, the orexinergic projections to the cholinergic and non-cholinergic neurons of the basal forebrain represent a critical pathway modulating the attention (Fadel and Burk, 2010). Systemic and intrabasalis administration of orexin-1 receptor antagonist can impair the sustained attention performance in rats (Boschen et al., 2009). The activity in the suprachiasmatic area was revealed reduced during a sustained attention task with increasing homeostatic sleep pressure (Schmidt et al., 2009). These findings indicate that the hypothalamus acts as a key hub of the attentional networks, and disruption of hypothalamic circuits could contribute to attentional deficits. Therefore, we speculate that the hypothalamic activity would be involved in the declined attentional performance after sleep deprivation.

Resting-state functional magnetic resonance imaging (rs-fMRI) is a non-invasive method to measure the intrinsic brain fluctuations based on blood oxygen level-dependent (BOLD) signals. Functional connectivity measures the temporal correlations between spatially separated regions, allowing to explore the neural connectivity under conditions of interest (Chee and Zhou, 2019). Previous rs-fMRI studies have demonstrated that altered activation in the frontoparietal networks, salience networks, default mode networks, and thalamic circuits are associated with attentional impairments following sleep deprivation (Ma et al., 2015; Krause et al., 2017). Nevertheless, the functional involvement of the hypothalamus in attentional performance after sleep deprivation has yet to be established.

Given its susceptibility to sleep deprivation and critical role in attentional control, we hypothesized that hypothalamic circuits would be relevant to declined attentional performance after sleep deprivation. To test our hypothesis, we compared the hypothalamic functional connectivity derived from rs-fMRI of 30 adult males between rested wakefulness (RW) state and 36 h of total sleep deprivation (TSD). Furthermore, the association between altered hypothalamic functional connectivity and vigilant attention performance was examined.

## Materials and Methods

### Participants

A total of 30 healthy male participants (aged 20–30 years, right-handed) were recruited in the study. The study was approved by the Institutional Review Board of Beihang University (Beijing, China). The study protocol was conducted according to the Declaration of Helsinki. All participants provided written informed consent prior to enrollment. After enrollment, participants received standardized medical examinations by specialist physicians, including interviews, questionnaires, physical examinations, electrocardiograms, and blood tests, to exclude acute or chronic medical and psychological conditions. The inclusion criteria were as follows: (a) have a regular sleep schedule (Pittsburgh Sleep Quality Index scores <7); (b) no history of cardiovascular, respiratory, nervous system diseases, psychiatric disorders, or sleep disorders; (c) non-smokers and non-alcoholics. Participants were informed to maintain regular sleep schedules as usual and keep a sleep diary in the week before the laboratory study. Participants were forbidden to take medication and consume stimulant drinks and food, such as caffeine, tobacco, alcohol, or tea within 1 week before and throughout the experiment.

### Experiment Procedure

The experiment was conducted in a sleep laboratory, with medical professionals present throughout the experiment course. Participants underwent rs-fMRI scans twice, once in RW state and once after 36 h of TSD. The two scans were performed with an interval of at least 3 weeks to limit the residual effects of TSD on cognitive function. Each participant was distributed a body-movement watch to monitor sleep-wake periods across the entire experiment. Participants arrived at the laboratory 1 day before the TSD session to adapt to the lab environment, with at least 8 h of sleep from 22:00 to 8:00. The TSD session started at 8:00 on the second day and ended at 20:00 on the third day, during which participants were monitored by experimental operators to remain awake. For the RW session, participants kept a normal sleep (from 22:00 to 8:00) after one night of adaptive sleep in the sleep laboratory. During waking state, participants were allowed to do some moderate activities, such as playing games, talking, reading, sitting for rest, and eating. The rs-fMRI scans were performed at the same timepoint (20:00) on the third day in both the RW and TSD sessions. The light was maintained on during the TSD session and fMRI scans while maintained off from 22:00 to 8:00 in the RW session.

### Resting-State Functional Magnetic Resonance Imaging Acquisition

The rs-fMRI scans were performed in a 3.0 T Siemens Magnetom Skyra scanner (Siemens Medical Solutions, Erlangen, Germany) using echo-planar imaging (EPI)-BOLD sequence. The scan parameters were listed as follows: repetition time = 2 s, echo time = 30 ms, flip angle = 90°, field of view = 256 mm × 256 mm, matrix size = 64 × 64, number of slices = 35, slice thickness = 3 mm, slice gap = 1 mm. The scans lasted for 480 s. Participants were instructed to lie supine in the scanner and hold their bodies and heads still throughout the scan. Their heads were comfortably restrained to reduce head movement. Earplugs were used to limit the potential effects of scanner noise. Before each scan started, the experimental operators used a microphone to remind participants to keep awake and not to think about anything during the scan.

### Resting-State Functional Magnetic Resonance Imaging Preprocessing

The rs-fMRI images were preprocessed using the DPABI software (Yan et al., 2016) based on MATLAB 2020a. The preprocessing procedure included the following steps: (1) removing the first 10 time points; (2) slice time correction; (3) realignment; (4) spatial normalization to the Montreal Neurological Institute (MNI) space at 3 mm × 3 mm × 3 mm; (5) regressing out nuisance covariates, including the Friston 24 head motion parameters, white matter signals, cerebrospinal fluid signals, and global mean signals; (6) spatial smoothing with a 5 mm full width half maximum Gaussian kernel; (7) band-pass temporal filtering (0.01 Hz -0.08 Hz) to reduce high-frequency noises (Yin et al., 2018). During data preprocessing, three participants were excluded due to impaired imaging data, and one participant was excluded due to mean frame-wise displacements (FD) larger than 0.5 mm. There was no significant difference in mean FD between TSD and RW states (paired *t*-test, 0.10 ± 0.05 vs. 0.11 ± 0.04, *P* = 0.394).

### Functional Connectivity Analysis

**FIGURE 1:**
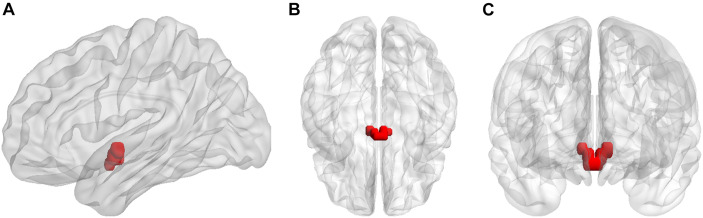
Seed regions for the hypothalamus in **(A)** sagittal, **(B)** axial, and **(C)** coronal views.

The left hypothalamus and right hypothalamus were defined as seed regions. The hypothalamic regions of interest (ROIs) were generated using the WFU PickAtlas software (Maldjian et al., 2003, 2004; Figure 1). Whole-brain functional connectivity patterns of the hypothalamus for each participant in both RW and TSD states were acquired via seed-based analysis using the DPABI software. First, the mean time series across all voxels within each ROI were calculated. Then, Pearson correlation coefficients were calculated between the mean time series of each ROI and the time series of each voxel in the rest of the brain. Fisher r-to-z transformation was applied to correlation coefficients to improve normality for analyses.

### Vigilant Attention Measurement

The psychomotor vigilance test (PVT) has been widely used to measure the sustained or vigilant attention. It has a high sensitivity to sleep deprivation and is documented to be free of aptitude and practice effects (Basner et al., 2018). The visual stimuli were presented as red dots on a liquid crystal display screen. The red dots appeared in succession with random intervals, with each lasted for 1000 ms and disappeared immediately after participants responded. Participants were requested to respond as quickly as possible by pressing the button of a keyboard once the red dots appeared. Response time (RT) for each stimulus was recorded and RT greater than 500 ms was defined as a lapse. We calculated the mean RT (average RT for all stimuli), fastest 10% RT (average RT for the fastest 10% stimuli), and lapse probability (number of lapses divided by the total number of stimuli) for each participant in both RW and TSD states.

### Statistical Analysis

The mean RT, fastest 10% RT, and lapse probability of PVT were analyzed using GraphPad Prism 7 software. The differences of PVT data between the TSD and RW states were normally distributed. We compared PVT performance between two states using a paired *t*-test. *P* < 0.05 was considered statistically significant in the analysis of PVT data. Whole-brain functional connectivity patterns for each seed region were acquired using a one-sample *t*-test in a voxel-wise manner. To investigate the alterations in hypothalamic functional connectivity after TSD, a paired *t*-test between TSD and RW states was carried out using the DPABI software. We used AlphaSim correction for multiple comparisons in both one-sample *t*-test and paired *t*-test in functional connectivity analysis, with the significance threshold set at *P* < 0.001 in a voxel-level and *P* < 0.01 in a cluster level (Ledberg et al., 1998; Liu et al., 2018). Furthermore, the Pearson correlation analysis was conducted to explore the association between altered hypothalamic functional connectivity and changed PVT performance following TSD. Bonferroni correction was applied in correlation analysis with a statistical significance level of *P* < 0.05.

## Results

### Demographic Features and Psychomotor Vigilance Test Performance

Among 30 recruited participants, three were excluded due to impaired imaging data, and one was excluded owing to excessive head motion. Therefore, twenty-six male participants aged (24.7 ± 2.7) years were included in the final analysis. Their body mass index was (23.7 ± 1.8) kg/m^2^. Compared to RW state, participants in TSD state had significantly longer mean RT (*P* = 0.018), longer fastest 10% RT (*P* = 0.007), and higher lapse probability (*P* = 0.006) (Table 1).

**TABLE 1:** Comparison of PVT performance between RW and TSD states.

	RW	TSD	*P*
Mean response time (ms)	355.6 ± 30.1	367.2 ± 34.3	0.018
Fastest 10% response time (ms)	282.3 ± 28.0	301.2 ± 37.2	0.007
Lapse probability (%)	8.9 ± 9.5	18.9 ± 16.4	0.006

*PVT, psychomotor vigilance test; RW, rested wakefulness; TSD, total sleep deprivation.*

### Whole-Brain Functional Connectivity Patterns of Hypothalamus

**FIGURE 2:**
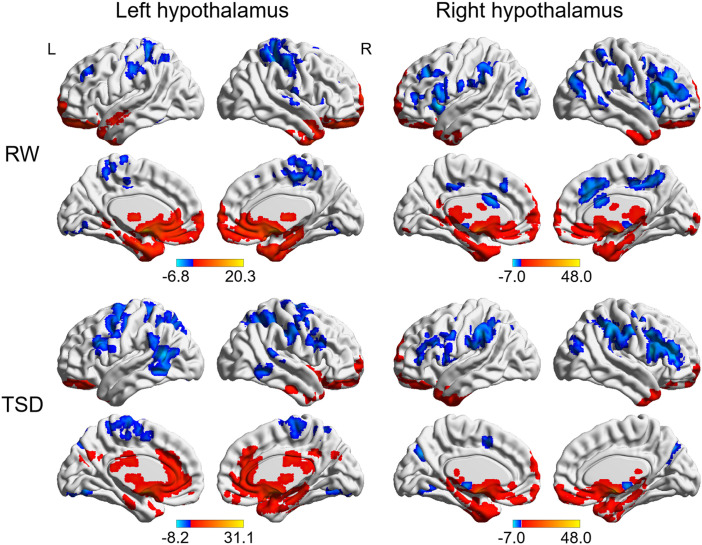
Whole-brain functional connectivity patterns of hypothalamus in RW and TSD states (AlphaSim corrected, *P* < 0.01). Colored bars indicate *t-*values of one-sample *t*-test within RW and TSD groups. RW, rested wakefulness; TSD, total sleep deprivation.

The whole-brain functional connectivity patterns for the left and right hypothalamus are shown in Figure 2. The bilateral hypothalamus showed similar functional connectivity patterns, which were qualitatively similar in RW and TSD states. Generally, the hypothalamus demonstrated positive functional connectivity with the orbitofrontal cortex, ventromedial frontal cortex, temporal pole, medial temporal lobe, cerebellum, midbrain, limbic regions, such as cingulate cortex, parahippocampal gyrus, hippocampus, insula, and amygdala, basal ganglia regions, such as the lentiform nucleus and caudate nucleus, as well as thalamus (AlphaSim corrected, *P* < 0.01). In contrast, the hypothalamus demonstrated negative functional connectivity with the lingual gyrus, superior temporal gyrus, middle frontal gyrus (MFG), superior frontal gyrus, sensorimotor cortex, and parietal regions (AlphaSim corrected, *P* < 0.01).

### Altered Hypothalamic Functional Connectivity After Total Sleep Deprivation

**FIGURE 3:**
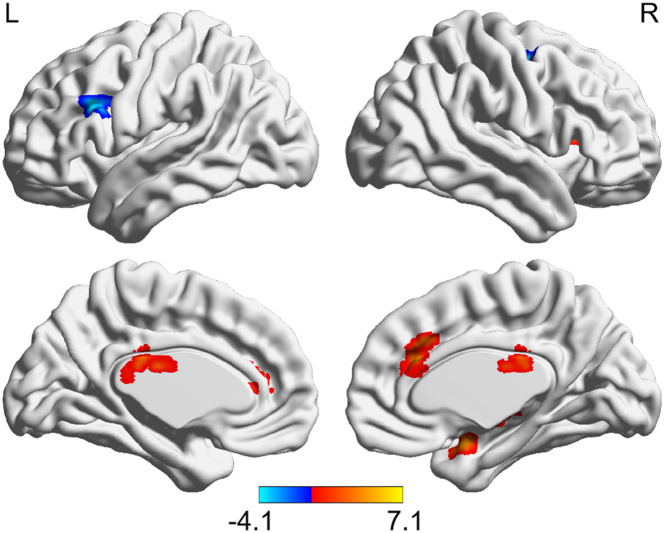
Altered functional connectivity of left hypothalamus after TSD (paired *t*-test, AlphaSim corrected, *P* < 0.01). Colored bars indicate *t*-values of paired *t*-test between RW and TSD states. RW, rested wakefulness; TSD, total sleep deprivation.

After TSD, significantly enhanced functional connectivity was observed between the left hypothalamus and right amygdala, right insula, bilateral thalamus, and bilateral anterior cingulate cortex (ACC), while reduced functional connectivity was observed between the left hypothalamus and bilateral MFG (AlphaSim corrected, *P* < 0.01, cluster size > 34) (Figure 3 and Table 2). However, significantly altered functional connectivity of right hypothalamus was not observed.

**TABLE 2:** Brain regions with significantly altered functional connectivity with hypothalamus after TSD.

Brain regions	*X*	*Y*	*Z*	Cluster size	*t*
**Left hypothalamus**					
Right amygdala	30	3	−21	40	4.62
Right insula	33	18	15	58	7.11
Left anterior cingulate cortex	−18	39	9	79	5.26
Right anterior cingulate cortex	9	30	24	68	4.67
Right thalamus	9	−9	−6	72	5.05
Left thalamus	−3	−15	21	41	4.66
Left middle frontal gyrus	−42	21	27	36	−4.07
Right middle frontal gyrus	30	−3	54	42	−3.72

*TSD, total sleep deprivation.*

### Correlation Between Altered Hypothalamic Functional Connectivity and Psychomotor Vigilance Test Performance After Total Sleep Deprivation

Furthermore, we performed Pearson correlation analysis between altered hypothalamic functional connectivity and PVT performance following TSD. However, no significant correlation survived after Bonferroni correction (all *P* > 0.05).

## Discussion

In the current study, we compared the hypothalamic functional connectivity derived from rs-fMRI between TSD and RW states. This is the first study to investigate the functional involvement of the hypothalamus in attentional impairments after TSD. We found that TSD induced increased functional connectivity between the left hypothalamus and bilateral ACC, bilateral thalamus, right amygdala, and right insula. Decreased functional connectivity was identified between left hypothalamus and bilateral MFG. Our results may provide new insight into neural signatures for attentional impairments elicited by TSD although a significant correlation between altered hypothalamic functional connectivity and changed PVT performance was not observed, which could be due to the small sample size of the study.

Compared to the RW state, the thalamus demonstrated increased functional connectivity with the hypothalamus after TSD. The thalamus has extensive connections with the cerebral cortex and modulates the cortico-cortical communication, controlling attention, consciousness, and sleep-wake transitions (Steriade and Llinas, 1988; Halassa et al., 2014). The thalamocortical functional connectivity exhibits a significant reduction following TSD, which may underlie the declined alertness after sleep restriction (Shao et al., 2013). Moreover, the aberrant thalamic activity is associated with cognitive impairments consequent to sleep deprivation, such as sustained attention, short-term memory, and learning (Yoo et al., 2007; Chee and Tan, 2010; Li et al., 2017). Previous studies found that lateral hypothalamic activity can directly modulate the thalamocortical networks, thus controlling the vigilant states. The activation of the hypothalamus-thalamic circuits can promote arousal (Herrera et al., 2016). Our results revealed increased functional connectivity between the hypothalamus and thalamus after TSD, suggesting that the brain augmented the communication within arousal networks to maintain alertness after acute sleep loss.

We also observed increased functional connectivity between the hypothalamus and bilateral ACC. ACC is involved in multiple brain functions, such as sleep regulation, consciousness state, conscious sensory perception, and efficient executive control (Piantoni et al., 2013). Accumulating neuroimaging studies have demonstrated that the cingulate cortex was implicated in the vigilant attention impairments induced by sleep deprivation. For instance, the cingulate activity exhibits a marked decrease during attentional tasks under the sleep-deprived state (Gujar et al., 2014). An electroencephalography study found that sleep deprivation impaired the effective functional connectivity from posterior to anterior cingulate cortex, which can predict the individual differences in the performance of a vigilant attention task after sleep restriction (Piantoni et al., 2013). Besides, enhanced anterior cingulate functional connectivity with cortical and subcortical regions, including frontoparietal areas, putamen, and anterior insula were revealed after one night of sleep deprivation (Fang et al., 2015). Indeed, ACC receives abundant cholinergic projections from the basal forebrain, thereby mediating arousal and attention (Swann et al., 2020). Moreover, orexinergic inputs from the lateral hypothalamus onto the basal forebrain represent a critical pathway in attentional processing (Fadel and Burk, 2010). Therefore, the hypothalamus may indirectly innervate the ACC activity in the regulation of attention. Collectively, the present study may indicate a compensatory increase of attentional network activity in an attempt to counter the declined attention following TSD. However, no significant correlation between altered hypothalamus-ACC connectivity and PVT performance was observed.

Besides, an increased functional connectivity was also identified between the hypothalamus and insula. The insula is a core hub of the salience network (SAN) and is involved in saliency detection, decision making, sensory processing, and attention regulation (Naqvi and Bechara, 2010; Cauda et al., 2011; Uddin, 2015). It is well documented that the insula plays an important role in the dynamic switching between other large-scale networks to facilitate bottom-up access to attentional resources, with which the brain maintains attention on external stimuli and makes them additionally significant and salient (Sridharan et al., 2008; Menon and Uddin, 2010; Liang et al., 2016). Multimodal neuroimaging studies have demonstrated that the insula was susceptible to sleep loss. The insula shows increased regional brain activity (Chen et al., 2018), increased gray matter volume (Long et al., 2020), and elevated cerebral blood flow (Elvsashagen et al., 2019) following sleep deprivation. A rs-fMRI study found increased functional coupling between SAN and default mode networks following sleep deprivation, suggesting upregulated assignment of saliency to internal mental events for survival (Lei et al., 2015). Our findings may reflect the upregulated bottom-up modulation from the hypothalamus to cortical attentional networks mediated by the insula to support attention under the sleep-deprived state.

We observed that right amygdala exhibited enhanced functional connectivity with the hypothalamus following TSD. Previous studies have demonstrated that sleep deprivation induced disrupted amygdala functional connectivity, which may underlie psychophysiology after sleep restriction. It is well documented that the amygdala is an essential area responsible for emotion processing, which is involved in the emotional changes after acute sleep deprivation (Shao et al., 2014). Moreover, the amygdala also plays an important role in attentional modulation since it has rich connections with the sensory networks and has indirect connections with the attention and arousal networks (Sarter et al., 2005; Abivardi and Bach, 2017; Pruessner et al., 2020). Close functional coupling between the hypothalamus and amygdala in attentional processing has been identified. The amygdala central nucleus is a prime target receiving the projections from the lateral hypothalamus, especially the orexin neurons (Ciriello et al., 2003). Lesions of rat lateral hypothalamus damage the amygdala-dependent attentional performance in associative learning (Wheeler et al., 2014). Our results suggest that the brain mobilizes additional attentional resources to meet the cognitive demands under increased sleep pressure. However, this redistribution of attentional resources is obviously not enough to counter the negative effects of sleep deprivation.

Another main finding is that functional connectivity between the hypothalamus and bilateral MFG was diminished following acute sleep loss. The prefrontal cortex is engaged in a wide range of cognitive processes, especially vigilant regulation and top-down attentional control (Gaggioni et al., 2018). The MFG is a key hub that mediates the interaction between dorsal and ventral attentional networks, maintaining the integrity of attentional neurocircuits. It plays a critical role in the dynamic network changes involved in attentional processing, thereby exerting a regulatory effect on vigilant attention (Song et al., 2019). Optimal performance of PVT was associated with greater activation in the frontoparietal sustained-attention network, which may reflect top-down modulation of attention required for the task (Drummond et al., 2005). Previous functional imaging studies demonstrated that prefrontal activity was associated with impaired sustained attention following sleep deprivation (Borragan et al., 2019). In this study, the decreased hypothalamus-prefrontal communication may indicate that the top-down attentional regulation is disrupted, leading to the declined vigilant attention after TSD.

The study featured several limitations. First, only male participants were recruited in the analysis, which would weaken the generalization of our results. Second, the study had a relatively small sample size. A larger sample size is needed for future researches to verify the current findings. Third, we did not objectively monitor the real-time sleep state during resting-state scans. In further studies, electroencephalography monitoring or eye tracking is necessary to exclude the interference of sleep. Fourth, AlphaSim correction was applied for multiple comparisons. Therefore, the result of the present study should be interpreted cautiously. Finally, the hypothalamus is a functionally heterogeneous region that has different subregions. It is involved in multiple physiological functions, such as temperature regulation, glucose metabolism, body fluid balance, ingestion, sleep/wake homeostasis, and attention processes. Whether alterations in physiological functions mediated the attention performance after TSD is an interesting issue to explore in further researches. Besides, future studies should focus on the alterations in functional connectivity of hypothalamic subregions after sleep deprivation.

## Conclusion

This study reveals significant differences in hypothalamic functional connectivity between RW and TSD states. Significantly altered functional connectivity is distributed in brain regions associated with arousal and attention regulation. We observed enhanced functional connectivity between the hypothalamus and thalamus, ACC, insula, and amygdala, while the reduced functional connectivity between the hypothalamus and MFG. The disrupted hypothalamic circuits may be implicated in the attentional performance following TSD, which extends the available evidence on neural mechanisms underlying the cognitive impairments induced by sleep deprivation.

## Data Availability Statement

The datasets generated for this study are available on request to the corresponding author. Requests to access these datasets should be directed to XZ, smrc301@163.com.

## Ethics Statement

The studies involving human participants were reviewed and approved by the Institutional Review Board of Beihang University (Beijing, China). The patients/participants provided their written informed consent to participate in this study.

## Author Contributions

JQ analyzed the data and wrote the first draft of the manuscript. B-ZL, YZ, and BP performed the experiment and collected the data. Y-HG and HZ contributed to the conception and design of the study. YL analyzed the data. Y-CS and XZ contributed to the conception and design of the study, and revised the manuscript. All authors approved the final version to be submitted.

## Conflict of Interest

The authors declare that the research was conducted in the absence of any commercial or financial relationships that could be construed as a potential conflict of interest.

## Publisher’s Note

All claims expressed in this article are solely those of the authors and do not necessarily represent those of their affiliated organizations, or those of the publisher, the editors and the reviewers. Any product that may be evaluated in this article, or claim that may be made by its manufacturer, is not guaranteed or endorsed by the publisher.
